# Can Mixed Parasite Infections Thwart Targeted Malaria Elimination Program in India?

**DOI:** 10.1155/2017/2847548

**Published:** 2017-08-16

**Authors:** Upasana Shyamsunder Singh, Nisha Siwal, Veena Pande, Aparup Das

**Affiliations:** ^1^Division of Genomic Epidemiology, ICMR-Centre for Research in Medical Entomology, No. 4, Sarojini Street, Chinna Chokkikulam, Madurai 625002, India; ^2^Department of Biotechnology, Kumaun University, Nainital 263001, India

## Abstract

India is highly endemic to malaria with prevalence of all five species of human malaria parasites of* Plasmodium* genus. India is set for malaria elimination by 2030. Since cases of mixed* Plasmodium* species infections remain usually undetected but cause huge disease burden, in order to understand the distributional prevalence of both monospecies infections and mixed species infections in India, we collated published data on the differential infection incidences of the five different malaria parasites based on PCR diagnostic assay. About 11% of total cases were due to mixed species infection. Among several interesting observations on both single and mixed parasitic infections, incidences of* Plasmodium falciparum* monoinfection were found to be significantly higher than* P. vivax* monoinfection. Also,* P. malariae* seems to be emerging as a potential malaria threat in India. Putting all the facts together, it appears that the dream of achieving malaria elimination in India will not be completely successful without dealing with mixed species infection.

## 1. Introduction

Humans simultaneously infected by different parasites are quite natural and frequent [1]. Around 14,000 pathogens have been described in humans [2] and approximately 30% of infections may be mixed infections or coinfections, although this can rise up to 80% in some human populations [3]. By coinfecting a host, parasites form a coevolutionary network [4] which can alter host susceptibility to other parasites, infection duration, transmission risks, and clinical symptoms. This condition poses potential threat to human health in treatment and prevention of the disease. Also, competition may occur between two or more parasite species of the same genus because they occupy the same physical space within their host [5]. In this scenario, gene exchange may take place via recombination or plasmid transfer that could be a potent driver of pathogen evolution, consequently resulting in functional changes which make the parasites highly virulent or resistant to drugs [6, 7].

To this extent, malaria is an age-old disease of the human kind, mainly restricted to the tropical and subtropical countries of the globe [8]. Several efforts with basic, applied, and operational research across the globe have not yet been successful in completely controlling malaria, although malaria infection has been severely curtailed by intervention through vector control and chemotherapy [9]. However, evolution and spread of drug-resistant, highly virulent parasites and insecticide-resistant mosquitoes have put new challenges in curtailing malaria to a significant extent [10]. Furthermore, due to large usage of improved surveillance and sensitive diagnosis tools in recent years, it is now easier to detect infections (either with single or with multiple species of human malaria parasites) in a single individual than before [11]. To this extent, the PCR diagnostic assay has evolved over the last decades as the principal and highly sensitive tool for malaria diagnosis in the laboratory [12]. This is because researchers from almost every malaria endemic country have utilized this diagnostic tool to determine the cases of single parasitic as well as mixed parasitic infections in a single individual in the laboratory setting.

India is highly endemic to malaria and one of the few countries contributing significantly to global deaths and morbidity due to malaria infection [13]. In 2015, according to estimate by the World Health Organization, about 140 million Indians were infected and there were 384 deaths due to malaria [14]. Varied and changing climatic conditions, urbanisation, global migration, man-made developmental changes, irrigation, and so forth, along with presence of all the five different species of* Plasmodium* and abundant distribution of nine species of Anopheles that are vectors of malaria parasites, have resulted in bringing out complex malaria in India [15]. Like in many endemic countries, laboratory diagnosis of malaria for epidemiological studies has taken a giant leap from microscopy to PCR diagnostic assay in India. As a result of this, enormous amounts of data on monoparasitic infections and mixed parasitic infections have been accumulated over the past decade across different malaria endemic provinces of India. Considering India is aiming to eliminate malaria by 2030 [16], as a first step to achieve this goal, it is of importance to understand abundance and differential distribution by both monoinfections and mixed infections of five different species of malaria parasites infecting human (*Plasmodium falciparum, P. vivax, P. malariae, P. ovale*, and* P. knowlesi*). Therefore, (i) in order to determine distributional abundance of mono-malaria parasite infections as well as mixed malaria parasite infections in all of India in a single communication, (ii) to generate debate on the probable cause of differential interactions of two or more different species of* Plasmodium* infecting a single individual at a time, and (iii) to synthesize if the current knowledge would be useful for the targeted malaria elimination program in India, we have collated published data (published between 2005 and 2017) on mono-malaria infections and mixed malaria infections based on PCR diagnosis in India [17–34] and present herewith comprehensive information that could be of potential interest to malaria public health in terms of targeted malaria elimination in India.

## 2. Uprising of* P. falciparum*, Range Expansion of* P. malariae*, and Abundance of Mixed Species Malaria Infection

**Figure 1:**
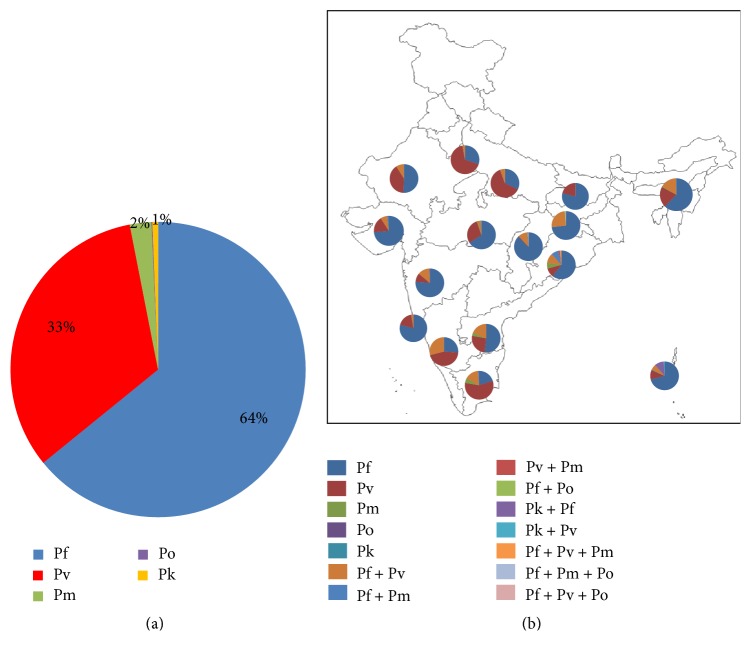
Malaria burden due to infection of different malaria parasites either as single or as mixed infection in India. (a) Pie chart showing contribution of each of the five human malaria parasite species (both monospecies infections and mixed species infections have been considered) into the net malaria burden in India. Clearly,* P. falciparum* has surpassed* P. vivax* and* P. malariae* is increasing its abundance in India. (b) Pie chart of infections with either mono-malaria parasites or different combinations of mixed malaria parasites in different Indian states (keys of different colour codes are given in the figure legend).

Collation of data from 19 publications involving 7821 malaria positive individuals sampled in 67 different geographic locations of 18 different Indian states at different time points (Table 1) reveals several interesting findings. If only the burden of infections by five different malaria parasites is considered (irrespective of their single or mixed infections), majorities were found to be due to* P. falciparum* (64%), followed by* P. vivax* (33%). The other three species have meagre contribution (*P. malariae*, 2%;* P. knowlesi*, 0.9%; and* P. ovale* 0.1%) to malaria infection in India (Figure 1(a)). If the extent of monoinfection across different malaria endemic Indian states is considered,* P. falciparum* was highest in Chhattisgarh (87%) followed by Goa (79%) and lowest in Tamil Nadu (20%) (Figure 1(b)). Similarly, highest monoinfection by* P. vivax* was evident in Delhi (66%) followed by Uttar Pradesh (62%) and the lowest one was in Chhattisgarh (0.33%) with no cases in Jharkhand (Figure 1(b)). Interestingly, monoinfection by* P. malariae* could be detected in five states with highest abundance in Odisha (6.4%) followed by Tamil Nadu (5.3%), Andhra Pradesh (3.3%), Madhya Pradesh (1%), and Asom (0.7%). However, monoinfections of* P. ovale* were found only in two individuals from Chhattisgarh, and* P. knowlesi* was restricted to Andaman and Nicobar only in three cases (Figure 1(b)). It is therefore evident that monoinfections due to* P. falciparum* have surpassed* P. vivax* in India. About a decade ago, the proportion of* P. falciparum* to* P. vivax* was about 50 : 50 [15], but at present this turns out to be 73 : 27. To be noted that Indian* P. falciparum* is resistant to chloroquine and other sulpha drugs [35]. Furthermore, large number of asymptomatic* P. falciparum* infections circulating in local populations in different endemic states of India [36, 37] and failure in efficacy of the most effective and dependable antimalarial, ACT (artemisinin combined therapy) have been reported in several Indian locations [38, 39]. Therefore, management of rising monoinfection by* P. falciparum* in India seems to be quite an uphill task. Considering alone the upsurge of* P. falciparum* monoinfection, the situation is quite alarming in terms of malaria public health in India.

**Figure 2:**
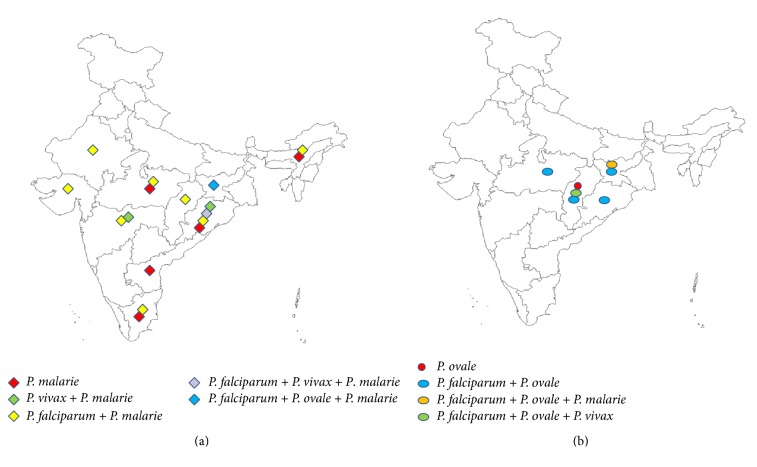
Distributional prevalence of the two less prevalent malaria parasites (*P. malariae* and* P. ovale*) in India. (a) To be noted is that* P. malariae* has been able to expand its range in almost all endemic locations in India, primarily as mixed infection with other species and principally in coinfection with* P. falciparum*. Odisha serves as the epicentre of every combination of diversity of coinfection by* P. malariae* with four other species of* Plasmodium* in India. (b) In contrast to* P. malariae*,* P. ovale* still has a very limited distribution, its capacity to coinfect with* P. falciparum* has helped in expanding its range in four different Indian states.

Malaria complexity in India is further compounded by the fact that all the five species of* Plasmodium* are often found to be in coinfection in a single individual. When data on mixed infections (in any combination among five different species) in a single individual are segregated based on different states, the incidence of mixed infection was found to be highest in Karnataka (30%), followed by Jharkhand (27%), Odisha (23%), Andhra Pradesh (18.5%), Andaman and Nicobar Islands (17.7%), Tamil Nadu (17%), North-eastern states (16.4%), Maharashtra (14%), Chhattisgarh (11.8%), Gujarat and Rajasthan (9%), Uttar Pradesh (5.6%), Madhya Pradesh (4.3%), Delhi (2.9%) and Goa (2.6%), with no cases of mixed infection in Bihar (Figure 1(b)). It is interesting to note that states presenting less number of monoinfection by* P. falciparum* (e.g., Karnataka) had the highest cases of mixed infection due to* P. vivax* and* P. falciparum*. Similarly, in Jharkhand where not a single case of* P. vivax* monoinfection was detected, 27% of mixed infections are due to* P. vivax* and* P. falciparum* coinfection. Apart from the mixed infection of* P. falciparum* and* P. vivax*, coinfections among other species of* Plasmodium* in India are quite infrequent with an exception of mixed infection involving* P. ovale* with either* P. vivax* or* P. knowlesi* in India. Among all mixed infection cases, one such case (between* P. malariae* and* P. falciparum*) needs significant attention. Mixed infection due to these two species was found to be distributed across several Indian locations, especially in the eastern and western parts (Figure 2(a)).* P. malariae* was once prevalent only in the eastern part of India (e.g., Odisha [40]) but has now expanded its range to the west also, that is, in Rajasthan and Gujarat (Figure 2(a)). This is possibly due to the fact that* P. malariae* can coinfect with* P. vivax* and* P. ovale*, though to a lesser extent than with* P. falciparum*. Since* P. vivax* is a well-adapted malaria parasite in India and endemic across Indian localities, the range expansion of* P. malariae* might have happened due to its ability to coinfect with* P. vivax* in India. Similarly, mixed infection due to* P. ovale* and* P. falciparum* which was reported in Odisha [41] has now expanded its range to central part of Indian states like Madhya Pradesh, Chhattisgarh, and Jharkhand (Figure 2(b)). It is interesting to note that all the diversities of mixed species infection of malaria parasites in possible combinations involving* P. falciparum*,* P. ovale*,* P. vivax,* and* P. malariae* are present in Odisha (Figures 2(a) and 2(b)). To be noted here is that both* P. malariae* and* P. ovale* are highly prevalent in the Thai-Myanmar border and in other Southeast Asian countries which are almost at the same latitude as that of Odisha [42–44]. Furthermore, Odisha had historical trade practice with Southeast Asian countries in the recent past. Therefore, observed similarities in patterns of mixed malaria parasite infections in Odisha and Southeast Asian countries are not surprising.

In order to understand if any defined trend of association exists in the occurrence of different monoinfections and mixed infections, it was interesting to note that* P. vivax* monoinfections was statistically significantly positively correlated (*r* = 0.8867; *P* < 0.00001) to* P. falciparum* across 18 Indian states. This indicates that Indian populations are susceptible for infection by both the parasite species and therefore explains the unique burden of both* P. vivax* and* P. falciparum* in India. This situation is opposite to Vanuatu where negative correlation in occurrence of these two species had been reported [45]. This situation is reflected by the fact that* P. falciparum* and* P. vivax* have different origins and they are placed far in phylogenetic tree as compared to the other human malaria parasites [46]. Interestingly, monoinfections of both the species were also found to be statistically significantly positively correlated to the mixed infection by both the species across Indian populations (*P. falciparum* monoinfection versus* P. falciparum* +* P. vivax*, *r* = 0.6646, *P* < 0.004;* P. vivax* monoinfection versus* P. vivax* +* P. falciparum*, *r* = 0.6722, *P* < 0.003). This means that mixed species infections are prevalent in both* P. vivax* endemic and* P. falciparum* endemic localities in India, thereby justifying that both of these parasite species have evolved greatly to coinfect Indians. Incidentally, both monoinfections and mixed infections by* P. malariae* were statistically strongly positively correlated with different combinations of species (*P. malariae* versus* P. falciparum* +* P. malariae*, *r* = 0.9537, *P* < 0.00001;* P. malariae* versus* P. vivax* +* P. malariae*, *r* = 0.8559, *P* < 0.0002;* P. falciparum* +* P. malariae* versus* P. vivax* +* P. malariae*, *r* = 0.9684, *P* < 0.00001). This indicates the adaptive affinity of* P. malariae* to coinfect with every other species of* Plasmodium* in a single individual in India. Interestingly, very few cases of coinfection involving* P. ovale* and* P. knowlesi* with other species could be found indicating their poor adaptive capacity in a coinfection state in a single human host in India.

## 3. Mixed Malaria Infection: Adaptive Evolution Favouring Parasitism?

**Figure 3:**
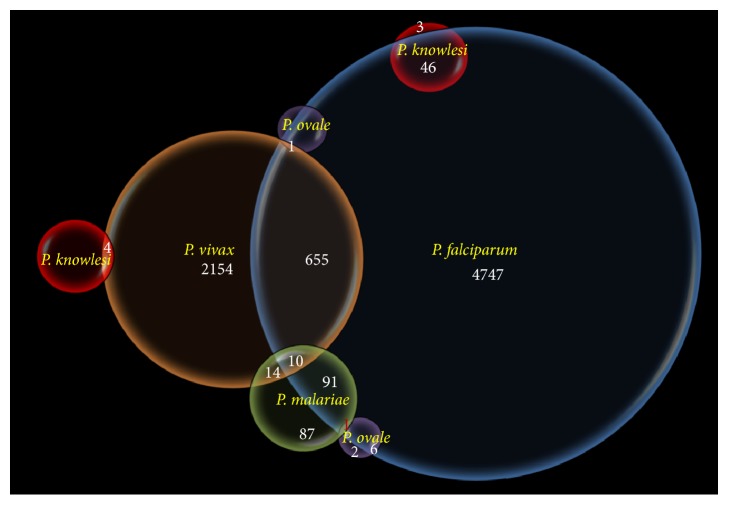
Venn diagram showing differential incidences and amount of interactions among five different species of malaria parasites in India. The numbers indicated against the name of the* Plasmodium* species are actual numbers of monoinfections and those that are indicated at each interaction are the number of mixed infections. Each circle is represented by a single species of* Plasmodium* and the size of the circles represents the relative prevalence of that species in India as evidenced through PCR diagnosis. To be noted here is that* P. falciparum* infections have grossly surpassed* P. vivax* infections in India.* P. knowlesi*, restricted so far to the Andaman and Nicobar Islands, is principally found as mixed infection either with* P. vivax* or with* P. falciparum*. Likewise,* P. malariae* is principally found as coinfection, that too majorly with* P. falciparum*.

**Figure 4:**
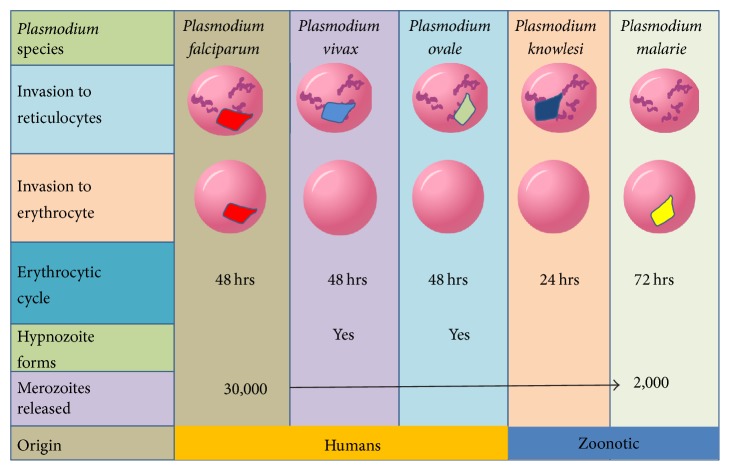
Host preference to different types of human Red Blood Cells (RBCs) by five different malaria parasites. To be noted is that, based on the common-niche-high-competition hypotheses, more incidences of mixed malaria parasitic infections were found in species that share dissimilar ecological niche (types of human RBCs).

The variable patterns of coinfection of five different species of the human malaria parasites in India (Figure 3) need to be discussed in terms of (i) preferential invasion of human Red Blood Cells (RBCs) of five different parasite species and (ii) intrahost competitive abilities. Firstly, the invasion preferences developed by human* Plasmodium* parasites have evolved to create a favourable environment for the parasites that provides abundant opportunity for transmission (Figure 4). It is often argued that degree of evolution in self-limiting techniques, such as preferential RBC invasion, is linked to the degree of concordant evolution of the parasite within its host [47]. Furthermore, wherever there is a strong preferential invasion of young RBCs, the maximum parasite replication rate has little impact on acute and chronic anaemia [47]. This is exemplified by the fact that* P. falciparum* has no selection for the type of RBCs for invasion (it invades both the young and matured RBCs, alike) (Figure 4), and that might be the reason for severe manifestation. On the other hand,* P. vivax*,* P. ovale*, and* P. malariae*, being selective for the type of RBC (Figure 4), cause less severe malaria [47]. Furthermore, preference of invasion of human reticulocytes by* P. knowlesi* merozoites has been suggested [48]. Competition may occur between two parasite species because they occupy the same physical space within their host [5]. Accordingly,* P. ovale* and* P. vivax* merozoites have similar preference for reticulocytes [47], which is why probably they do not coexist. If the parasites modify their respective ecological niches, they might in due course be able to coexist [49] and such cellular niche expansion may increase adaptive parasitism, which, in turn, may also increase pathogenesis. For example, it has been shown in vitro that* P. knowlesi*, which otherwise invaded all kinds of RBCs of its natural host macaque, can also invade a wider age range of human RBCs [50]. Considering the smallest lifecycle of only 24 hours of this species among all other species infecting human, if* P. knowlesi* evolves to invade all kinds of human RBCs, it might emerge as the most fatal of all the* Plasmodium* species infecting humans.

In a genetic viewpoint,* P. ovale* and* P. vivax* have similar breadth and sequence type of the* pir* (*Plasmodium* interspersed repeats) gene repertoires [51], which is the largest multigene family present in all* Plasmodium* genomes reported thus far [52]. Although the function of this protein family is not completely understood, it seems to promote parasite development in both the liver and blood, either by supporting parasite development within hepatocytes and erythrocytes and/or by manipulating the host immune response [53]. Since* P. malariae* contains only a restricted subset of the* pir* gene family [51] and almost 50% of* pir* genes in* P. malariae* are pseudogenes, host adaptation in* P. malariae* is less restricted than in* P. falciparum* [51]. This might probably explain why* P. malariae* is mostly found as mixed infection with other species of* Plasmodium* in India (Figure 3). Also, the* kelch13* gene of* P. falciparum* (associated with artemisinin resistance) is 84.8% and 82.7% similar to* P. malariae* and* P. ovale*, respectively [54]. Moreover, in the present dataset,* P. knowlesi* shows its prevalence more as a coinfection with* P. falciparum* than infecting as a single parasite. Since the three species (*P. knowlesi*,* P. malariae*, and* P. ovale*) are phylogenetically close to each other [55] and are also selective for specific types of RBC (Figure 4), it might be true that these three species of* Plasmodium* are in the learning phase and therefore seek help of an evolved parasite* (P. falciparum)* that can invade any kind of RBC (Figure 4) [47]. Therefore, malaria parasites are continuously evolving for better parasitism through their less-complex genomes [56] and less generation time, in comparison to their human or mosquito hosts. For example, considering the possibilities for regular genetic exchange within* P. malariae* populations [57], development of a selective inhibitor for* P. malariae* may prove more challenging than the development of one for* P. falciparum* [58].

Adaptation by Darwinian natural selection at both genetic and phenotypic level is evidenced in many species including malaria parasites in India [59]. Cases of nonrandom association between different parasites coinfecting a single host is also an indication of preferential (and therefore adaptive in nature) association. Some such incidences are worth mentioning here. For example,* P. ovale* was invariably found in association with* P. falciparum* and* P. malariae*, and not a single case of* P. ovale* to coinfect with* P. knowlesi* and* P. vivax* could be detected. This can be explained in terms of the fact that both* P. vivax* and* P. ovale* form hypnozoites (dormant parasite stages in the liver that cause relapse weeks to years after the primary infection [60]) therefore share a common niche. It is widely known that niche sharing leads to competition; thus, in order to avoid intrahost competition,* P. ovale* might not associate with* P. vivax* in a coinfection. The present epidemiological finding in India together with similar finding in other malaria endemic countries on coexistence of five different species at different combinations (Canada,* P. ovale* and* P. falciparum* [61]; China-Myanmar border,* P. malariae* and* P. ovale* [44]; Switzerland,* P. falciparum* and* P. malariae* [62]; and China,* P. knowlesi* and* P. falciparum* [63]) on the differential species infection in a single host can be discussed by the “hitchhiking” hypothesis [64]. Accordingly, when two different species coinfect a single host, one parasite species is unable to manipulate the host machinery, getting benefit from the manipulative potential of the other species [64]. The epidemiological finding on the coinfection of five different malaria parasites in India thus seems to follow the “hitchhiking” hypothesis, as the three otherwise less prevalent malaria parasites (*P. malariae*,* P. knowlesi* and* P. ovale*) mostly occur as coinfections mainly with* P. falciparum* and to certain extent with* P. vivax.*

## 4. Conclusions and Future Prospects

Clearly, India is heavily burdened with appreciable number of malaria cases with increasing number of* P. falciparum* monoinfections, and mixed* Plasmodium* species infections in a single individual. However, conclusions made out of the results presented in this communications suffer from several limitations. For example, (i) the PCR diagnostic assay has been performed in many different laboratories without following a uniform molecular biological protocol, (ii) the results of mixed infection came as a by-product of some other research work, and therefore sampling might have been biased, (iii) malaria samples have not been collected in defined period of year, and (iv) data from each and every malaria endemic locations have been included in the present analysis. Even then, the present dataset, prepared out of collation of published literature on mixed malaria infection, provides enough insight on the trends of increasing incidences of* P. falciparum* monoinfection and occurrence of mixed infection of five different malaria parasites in variable proportions.

Over several years, many agendas (either global or local) for either eradication or elimination of malaria have been prepared and implemented. The Global Malaria Eradication Program (GMEP), which ran from 1955 to 1969, had helped in reduction of malaria cases globally to a significant extent. However, technical challenges and resurgence of malaria in many countries including India led to a shift away from malaria elimination for many decades. Since 2000, there has been an increasing political drive to eliminate malaria [65], and reducing the burden of malaria is a central component of the Millennium Development Goals (MDGs) [65]. The current approaches to malaria elimination by the World Health Organization (WHO) have been fueled by the GMEP, which can crown a country to have achieved malaria elimination when there is reduction to zero of the incidence of infection caused by a specified malaria parasite in a defined geographical area as a result of deliberate efforts [66]. With a view to eliminate malaria in India, a national framework for malaria elimination in India (2016–2030) has been prepared which aims to eliminate malaria (zero indigenous cases) in the entire country by 2030. Accordingly, it is planned to eliminate malaria from 15 low-endemic states and Union Territories by 2020 and from moderate endemic states by 2022 and ultimately eliminating malaria from the whole country by 2027 and sustain zero transmission until the next three years to be officially declared by the WHO as a malaria-free country [67]. However, neither nationwide epidemiological information nor disease burden due to mixed species infection is available in India. The information presented here in one way throws enough light on malaria burden due to increasing* P. falciparum* monoinfection as well as mixed species infection and entails considerable risks that might come in the way of ambitious malaria elimination program in India. Considering (i) the fact that there is no defined diagnostic method for detection of mixed species infection at the local health centre, (ii) high species diversity of anopheline mosquitoes that are vectors to* Plasmodium* parasites, and (iii) unavailability of a defined treatment program for mixed species infection and increasing trends of drug-resistant parasites [68], goals set for malaria elimination seem far from achievement in India. Integrated efforts with in-depth surveillance for (i) malaria epidemiology specially focussing on increasing incidences of* P. falciparum* malaria and expanding range of* P. malariae*, (ii) type of infection (monospecies/mixed species), and (iii) distribution of drug and insecticide resistance malaria parasites and vectors, respectively [69, 70], would provide baseline data on which malaria elimination strategy could be assembled with effective case management and parallel control of local mosquito vectors. As of now, malaria elimination in India seems to be quite an uphill task.

## Figures and Tables

**Table 1:** Details of data on the differential prevalence of monoinfections and mixed infections by PCR diagnostic assay in India. To be noted is that the dataset consists of 67 different locations taken from 18 Indian states, retrieved from 19 different publications spanning from 2005 to 2017.

Sr. number	Name of state	Location and year (in parenthesis) of sample collection	Reference and number (in parenthesis)
(1)	*Delhi*	Nandnagri (2004) Shahdara (2004)	Nandwani et al. 2005 [17]
		Dwarka (2011–2013)	Singh et al. 2014 [18]
		Delhi (2014)	Unpublished data

(2)	*Asom*	Asom (2004–2006) Sonapur (2007) Tezpur (2011-2012) Diphu (2013) Guwahati (2013)	Keen et al. 2007 [19] Gupta et al. 2010 [20] Haanshuus et al. 2016 [21] Unpublished data Unpublished data
		Dibrugarh (2011-2012) Tinsukia (2011-2012) Karbi-Anglong (2011-2012) Chirang (2011-2012)	Sharma et al. 2013 [22]

(3)	*Arunachal Pradesh*	Rohit (2011-2012) Changlang (2011-2012)	Sharma et al. 2013 [22]

(4)	*Tripura*	Manu Bazar (2014)	Krishna et al. 2015 [23]
		Shantir Bazar (2014)	Krishna et al. 2015 [23]

(5)	*Uttar Pradesh*	Shankargarh (2015)	Unpublished data

(6)	*Rajasthan*	Bikaner (2007-2008)	Kochar et al. 2014 [24]
		Bikaner (2010-2011)	Nayak et al. 2013 [25]
		Udaipur (2014)	Krishna et al. 2015 [23]

(7)	*Bihar*	Raxaul (2011-2012)	Haanshuus et al. 2016 [21]

(8)	*Madhya Pradesh*	Madhya Pradesh (2004–2006)	Keen et al. 2007 [19]
		Shivpuri (2009) Dindori (2009)	Singh et al. 2010 [26]
		Balaghat (2010–2012) Jabalpur (2010–2012)	Patel et al. 2014 [27]
		Jabalpur (2008–2012)	Jain et al. 2013 [28]
		Balaghat (2012)	Bharti et al. 2013 [29]
		Betul (2012-2013)	Unpublished data
		Jhabua (2014)	Krishna et al. 2015 [23]
		Annupur (2014)	Krishna et al. 2015 [23]

(9)	*Jharkhand*	Jaldega (2014)	Krishna et al. 2015 [23]
		Bano (2014)	Krishna et al. 2015 [23]

(10)	*Gujarat*	Dahod (2014)	Krishna et al. 2015 [23]
		Valsad (2014)	Krishna et al. 2015 [23]
		Nadiad (2015)	Unpublished data

(11)	*Chhattisgarh*	Raipur (2007)	Gupta et al. 2010 [20]
		Bilaspur (2010)	Kumar et al. 2013 [32]
		Mungeli (2011-2012)	Haanshuus et al. 2016 [21]
		Jagdalpur (2014)	Krishna et al. 2015 [23]
		Baikunthpur (2014)	Krishna et al. 2015 [23]
		Bastar (2013-2014)	Chaturvedi et al. 2015 [30]

(12)	*Maharashtra*	Maharashtra (2004–2006)	Keen et al. 2007 [19]
		Ratnagiri (2011-12)	Haanshuus et al. 2016 [21]
		Gadchiroli (2012)	Unpublished data
		Gadchiroli (2014)	Krishna et al. 2015 [23]
		Gondia (2014)	Krishna et al. 2015 [23]

(13)	*Odisha*	Odisha (2004–2006)	Keen et al. 2007 [19]
		Rourkela (2008)	Gupta et al. 2010 [20]
		Mayurbhanj (2008) Sundergarh (2008) Keonjhar (2008) Nayagarh (2008) Rayagada (2008) Kalahandi (2008) Kandhamal (2008) Anugul (2008)	Dhangadamajhi et al. 2009 [31]
		Keonjhar (2012–2014)	Pati et al. (2017) [34]
		Mayurbhanj (2012–2014)	
		Keonjhar (2013)	Unpublished data
		Rourkela (2012)	Unpublished data
		Koraput (2014)	Krishna et al. 2015 [23]
		Rayagada (2014)	Krishna et al. 2015 [23]

(14)	*Andhra Pradesh*	Anantapur (2011-12)	Haanshuus et al. 2016 [21]

(15)	*Goa*	Goa (2004–06)	Keen et al. 2007 [19]
		Panaji (2008)	Gupta et al. 2010 [20]

(16)	*Karnataka*	Bengaluru (2008)	Gupta et al. 2010 [20]
		Mangalore (2014)	Unpublished data

(17)	*Tamil Nadu*	Chennai (2008)	Gupta et al. 2010 [20]
		Oddanchatram (2011-2012)	Haanshuus et al. 2016 [21]
		Ambur (2011-12)	Haanshuus et al. 2016 [21]
		Chennai (2014)	Unpublished data

(18)	*Andaman & Nicobar*	PortBlair (2004) Car Nicobar (2004) Teressa (2009) Campbell Bay (2010)	Tyagi et al. 2013 [33]
